# Transport Fitness of Cull Sows and Boars: A Comparison of Different Guidelines on Fitness for Transport

**DOI:** 10.3390/ani6120077

**Published:** 2016-11-28

**Authors:** Temple Grandin

**Affiliations:** Department of Animal Science, Colorado State University, Fort Collins, CO 80523, USA; Cheryl.miller@colostate.edu

**Keywords:** sows, boards, welfare, fitness for transport, lameness, codes of practices

## Abstract

**Simple Summary:**

Breeding sows and boars that are shipped to slaughter, auctions, or buying stations have a greater risk for welfare problems because they are older animals. Sows are sold when they fail to rebreed, are too thin or have difficulty walking. The transport guidelines of four organizations were compared. Most guidelines typically do not allow transport of non-ambulatory, severely injured animals or sows likely to give birth. The guidelines were less likely to agree on transport of extremely thin sows.

**Abstract:**

Sows and boars that have reached the end of their productive lives have a greater risk for welfare problems. This paper reviews literature on culling reasons that may affect the animals’ fitness for transport. The top two reasons identified for culling boars were: obesity and reproductive problems. Sows are most often culled due to lameness, low body condition, or failure to rebreed. The OIE (World Organization for Animal Health) fitness for transport guidelines that would apply to sows and boars were compared with documents from the Canadian Code of Practice, Northern American Meat Institute (NAMI), EU-UK-DEFRA (European Union-United Kingdom, Dept. Environment, Food and Rural Affairs), U.S. National Pork Board, European Practical Guidelines to Assess Fitness for Transport of Pigs, and U.S. Pork Trucker Quality Assurance. The guidelines had the greatest agreement on the following fitness for transport issues: non-ambulatory, severely injured animals, sows in the last ten percent of pregnancy and sows with uterine prolapses were not fit for transport. There was less agreement on low body condition. One of the reasons for the lack of agreement is that there were stakeholders who specialized in transporting and processing extremely thin animals. A standard that would severely restrict the transport and slaughter of these animals could hinder the business practices of these stakeholders. Many welfare specialists would agree that some of these animals would be unfit for transport.

## 1. Introduction

Numerous papers have been written that cover market pig welfare during transportation [1,2,3,4,5] but little attention has been given to cull sows and boar fitness to transport. Cull sow and boar handling and transport can be quite variable in that standard practices have not been adopted. With a lack of empirical data and variable practices, establishment of standards based on science is difficult albeit necessary. 

According to the United States Department of Agriculture (USDA), approximately 8000 to 10,000 sows and boars are sold in the United States and Canada daily [6]. These are animals that have reached the end of their useful breeding life. On a single business day, 15 April 2016, 700 old boars were marketed in North America [6]. In Europe, a Danish study showed that 400,000 sows are transported each year to slaughter [7]. Compared to young market weight pigs, the boars are a small fraction, but they are a market segment that has the potential to have severe animal welfare problems [8]. More research and information on cull sow and boar welfare after they leave the farm needs to be conducted because older animals may be more likely to have welfare problems. Therefore, the goal of this commentary is to cover the welfare literature that is available on cull sows and boars. A second objective is to compare existing industry guidelines on fitness for travel with the guidelines from the OIE [9]. Compared to young market weight pigs, welfare problems associated with fitness for travel are likely to increase in cull sows and boars that have reached the end of their productive life.

## 2. Reasons for Removing Boars and Sows from the Breeding Herd

An old study in Minnesota, U.S., a survey of 84 herds and a sample of 440 boars indicated that obesity was the major reason for culling 47% of the boars. The author has observed in the 1980s that some small producers made pets out of boars and overfed them. The other removals were for reproductive problems (18%), leg problems (12%), and death (7%) [10]. Even though this study is over 20 years old, more recent studies cited below show that lameness and reproductive problems are still major reasons for culling. A Norwegian study of sows housed in groups on plastic or concrete slats had a prevalence of 13% lame sows [11]. When floor hygiene was poor, lameness was 2.8 times higher. Another study showed the prevalence of lame sows ranges from 8.8% to 19.9% [12]. Inspection of the hooves at a large U.S. slaughter plant indicated that 21% of the sows had rear hoof overgrowth [13]. The front hooves were cracked on 22.6% of front feet and 18% of the rear feet [13]. Another study divided the herds into high and low performing. In high performing herds, there were greater numbers of boars culled for reproductive conditions [14]. A recent survey on four farms in Southern China recorded lameness as the major reason (35%) followed by reproductive issues at 20% [15]. Almost 20% of the sows were culled at their first parity [15]. The high cull rate of sows may be due to hot, humid conditions. This may have contributed to a lack of estrus expression by gilts. Another survey at two farms in Hungary indicated that 40% of the culls were due to leg problems and 51% were due to reproductive problems [16].

The majority of boars were culled for being overweight, but lactating sows often had problems maintaining body condition. Sows get culled for being lame or failing to rebreed [15,16]. Body condition is not usually used as a reason for culling but sows may enter the transportation and marketing channels that have poor body condition. A survey by researchers at Iowa State University at fifteen pig buying stations that purchase both market weight pigs and sows and boars showed that sows and boars were 86% of the non-ambulatory fatigued animals and 73% of the lame animals [17]. In the total population, which included market weight pigs, fatigued pigs equaled 16% and lame pigs only 5% [2]. Sows and boars were 82% of the animals with a body condition score of 1 and only 4% of the total population had a body condition score of 1 [17]. Unpublished U.S. industry data collected at eight different slaughter plants from an on-line survey of all cull swine both market weight and cull breeding stock reported that approximately 3% were emaciated. An animal was classified as emaciated if its ribs were clearly visible. The illustration in the AMI Recommended Animal Handling Guidelines and Audit Guide was used. Poor body condition is related to lower reproductive performance [18]. Sows need to have adequate body weight and condition after weaning their piglets to avoid being culled for failure to breed back [19]. A survey of 3158 cull sows in two U.S. slaughter plants indicated that low body condition was related to greater abnormalities of the ovaries [13].

Another study of 502 sows at a slaughter plant looked at the condition of a sow’s internal organs. At the farm, 50% of the sows were culled for reproductive problems. Seventy-five percent of the uteri were normal, 18% had signs of purulent infection, 62% positive for *E. coli* (*Esherichia coli*) and 52% inflamed [20]. The ovaries were normal in 54% of the sows [20]. Research is needed to develop improved management to reduce culling due to infections and inflammation. Potentially, a system should be developed so that producers would get feedback from the slaughter plant on the condition of the internal organs of their sows. The author’s opinion is that this would help producers improve production practices. 

It is beyond the scope of this paper to review and discuss how different housing systems or production practices affect the welfare of sows and boars. Production systems may have an effect on breeding animal longevity, which has the potential to either reduce or increase the numbers of breeding animals that enter the transportation system.

## 3. Marketing and Transport of Cull Sows and Boars in North America

There are numerous slaughter plants that process market weight pigs in both the U.S. and Canada. There are fewer plants that specialize in making sow meat products. This is relevant because transport distances may be increased. Market weight gilts and barrows are usually not processed in these facilities. As a service to producers, some companies that process market weight pigs will accept cull sows and then ship them to a sow facility for processing. Unfortunately, there are no published guidelines in the U.S. for the handling and transport procedures that are specific for sows and boars.

In North America, there is only one slaughter plant that processes cull boars. Some boars may pass through a complex marketing chain which involves numerous collection points due to a lack of slaughter facilities that will accept boars. The boars may have to be loaded and unloaded for more than one trip. It has been suggested that cull boars should be euthanized on farm [8]. This suggestion is likely to be criticized from a sustainability standpoint, because large amounts of edible meat would be wasted. To prevent both suffering and meat waste, sows and boars should be transported off the farm before they have deteriorated to the point that transport would cause suffering. The industry needs to develop methods for handling and transport that will maintain an adequate level of animal welfare. The next step in developing an improved system is to survey existing transport practices from the buying stations through the final destination—the slaughter plants.

Mature boars can seriously injure each other during fighting. In North America, it is a normal practice is to cut off the tusk ends to reduce injuries [21]. The percentage of boars that have their tusks cut is not known. In a study of 150 mature boars at an assembly yard in Manitoba, Canada the length of the tusks was not related to skin lesions due to fighting [22]. Canadian guidelines require that boars weighing over 135 kg be detusked prior to co-mingling [8]. The wording in the guideline is detusked [8]. It does not specify the length of the tusks.

Some codes of practice require mature boars to be transported in single separate compartments [8]. Both the author’s own observations at a mature boar slaughter facility and the survey in Manitoba, Canada indicated that aggression between mature boars is low if sows are not present. The author observed the behavior of mature cull boars in the lairage of a slaughter plant that was dedicated to mature boars. There were no sows on the premises. The boars were penned in groups and each boar had 4 m^2^. Extra space allowed the boars to move away from each other. Aggression between the boars was minimal. Therefore, the use of dedicated boar facilities that are never used for sows may help reduce aggression. Social factors can greatly influence aggression between pigs. Mixing a mature breeding boar in with younger market weight pigs reduced aggression [23]. This study shows that there may be novel methods to reduce aggression during marketing. In conclusion, there is very little published literature on the marketing process of mature boars, and it is an area that needs further study. 

## 4. Comparison of OIE Requirements with Other Cull Sow and Boar Welfare Guidelines

Table 1 outlines six different codes of practice and welfare assessments that would apply to the transportation of cull sow and cull breeding boars. These codes are compared to the OIE World Organization for Animal Health transport guidelines [9].

The guidelines reviewed are:

- Canadian Code of Practice [22];
- NAMI, A voluntary industry guideline published by the North American Meat Institute, which covers animal welfare during transport to the slaughter plant and all procedures inside the plant [18];
- UK and EU live transport: DEFRA [24] European regulations state that the veterinarian has a mandate to determine whether or not animals are fit to enter the food chain. If not, they have to be killed humanely;
- Common Swine Industry Audit [25]: This is a voluntary industry guideline published by the National Pork Board in the United States. It contains recommendations for conditions where euthanasia on the farm is recommended. These animals would not be fit for transport;
- UECBV [26] Practical Guidelines to assess fitness for transport of pigs. A European guide created by livestock industry, veterinary, and animal welfare groups. Council Regulation (EC) No. 1/2005 On the protection of animals during transport and related operations and amending directives 64/432/EEC and 93/119/EC Regulation (EC) No. 1255/97 [27];
- U.S. Transport Quality Assurance [28]. This guide has all the OIE fitness for transport guidance within it.

## 5. Discussion

### 5.1. Assessing Conditions that Make a Cull Sow or Boar Unfit for Transport

People who are working on farms need easy-to-use clear guidelines for determining whether or not an animal is fit for transport. Some of the guidelines outlined in Table 1 are easy to determine because the sow or boar either has the defect or does not. Some examples of clear guidance in the different guidelines are: a prolapsed uterus, broken leg, blind in both eyes, sows that are in the last 10% of pregnancy, had given birth within 48 h, or are unable to walk (non-ambulatory). In the UECBV guidelines, reversible rectal prolapses are allowed but uterine prolapses are not fit for transport. North American stakeholder agreed on not transporting sows with uterine prolapses. The author has participated in animal welfare panels for the OIE slaughter committee [29], North American Meat Institute (NAMI) and the American Veterinary Medical Association (AVMA) [30], and, from this experience, the aforementioned conditions were also easier for all stakeholders involved to agree on. There have been many conflicting opinions on body condition and lameness regarding how fit the animal is for transport. This is one of the reasons why many of the guidelines are vague. On the NAMI guideline BCS 1 was made a secondary criteria and in the OIE transport standard, the body condition score section is vague. Table 1 contains the exact wording and the OIE standard provides no further explanation. One of the reasons for this is that there are some stakeholders who either specialize in slaughtering thin sows or work in developing countries where protein from thin animals needs to be salvaged. In the OIE guidelines, it states those that cannot be moved without causing additional suffering. This is extremely vague and would be subject to many different interpretations.

### 5.2. Objective Outcome Measures to Make Assessment of Fitness to Transport Easier

Objective scoring systems have been very successful for improving animal welfare at slaughter plants [31]. Similar systems based on this model could be used for transport. Animal based measures such as percentage of animals falling, vocalizing during handling, or are moved without an electric goad are measured [25,32,33,34,35]. When these measurements are used, it is possible to determine if handling practices have either improved or deteriorated. In the U.S., the use of handling scoring has improved animal handling at slaughter plants [36]. Objective measures used to detect handling problems in pork slaughter houses are blood lactate, electric prod use, and certain types of skin lesions that are indicative of poor handling [34,36,37,38,39]. In Denmark, when carcasses are inspected for bruises, it may be possible to determine which ones were inflicted by people [36]. Blood lactate can be used to assess short-term stresses during the last five minutes before stunning. Electric prod use and pigs jamming in the stunning race is associated with higher lactate [38].

#### 5.2.1. Assessing Lameness

Lameness definitions in guidelines related to fitness for transport have been vague (Table 1). The author proposes the use of lameness scoring systems to help determine an animals’ fitness to transport. Scoring systems are available for assessing pig lameness [40]. A major problem is that people are using many different scoring systems. They range from a three-point scale used in Welfare Quality to a five-point scale used in the dairy industry (www.zinpro.com). A five-point scale for pigs has high inter-observer repeatability [41,42]. A two-point scale did not provide improved inter-observer reliability [42]. A study with sows suggested that fewer than five categories may improve interobserver reliability.

#### 5.2.2. Fitness for Transport: The Biggest Challenge!

The author has observed while serving on committees that write guidelines that, an inherent problem is getting all stakeholders to agree on a fitness for transport cutoff criteria points. It is the author’s opinion that there may be a need to develop guidelines for short or long trips. For sows and boars, this will require more research. Many of the guidelines in Table 1 agree that non-ambulatory animals that are unable to walk are not fit for transport. The question is what degree of lameness is fit for transport. Welfare experts agree that lameness is a major welfare issue [41]. The author has had frustrating discussions with government regulators who wish to avoid a clear guideline on welfare issues. They have made statements to the author such as “we need flexibility in enforcement” and that a clear guideline provides guidance they wish to avoid. Some government regulators actually appear to prefer vague guidelines.

If the author was given the task of determining which lame sows are either fit or not fit for transport, it would be done with a series of videos. Videos would be taken of many sows showing lameness ranging from mild lameness that would obviously be fit for transport to sows or boars that could barely walk. Veterinarians, welfare specialists, and stakeholders would rank the videos into fit and unfit for travel, to determine a consensus on a cutoff point. From this, a training video could be made for producers, truckers, and packers.

There are many factors that can affect animal welfare during transport such as temperature, stocking density, and animal condition [43,44,45,46,47]. For sows and boars, research is needed to determine how the animal’s condition before loading interacts with distance travelled, road conditions (smooth vs. rough), temperature, and other factors. Further research could also help determine what conditions are worsened by transport.

## 6. Conclusions

Handling and transport of cull breeding boars and sows is an area where more research is clearly needed. The author recommends doing a survey at slaughter plants that processes sows and boars to determine the condition of incoming animals. It should use the same methods as the slaughter surveys that have been conducted for cull dairy and beef cows [44]. Some boars are traveling long distances to a single North American slaughter facility. A major concern is determining fitness for transport. Many welfare issues that occur during transport are due to putting unfit animals on a vehicle [46,47]. Major stakeholders agree that downed non-ambulatory animals, animals with fractures, animals in the late stages of pregnancy, and sows with uterine prolapses should not be transported off the farm. Guidelines for fitness for transport based on body condition and lameness are vague because major stakeholders to not agree. One of the reasons for disagreement in North America is that there were stakeholders who specialized in processing thin sows. The main reason for the disagreements are that people’s views differ. The author has served on animal welfare committees for three different species. There are some producer stakeholders who want a weak standard that will enable poorer producers to pass an audit. Producers who have sows or boars arriving at the slaughter plant in poor condition should be given a reasonable time to improve the condition of their animals.

## Figures and Tables

**Table 1:** Comparison of OIE (World Organization for Animal Health) requirements on unfit to travel with other regulations and industry guidelines that would apply to sows and boars ^1^.

	OIE World Organization for Animal Health Transport of Animals by Land Exact Wording on Unfit to Travel	Canadian Code of Practice for Pigs	NAMI ^2^ Animal Handling Guidelines	UK and EU Live Transport DEFRA	Common Swine Industry Audit Based on Recommendation for On-Farm Euthanasia	UECBV Practical Guidelines to Assess Fitness for Transport of Pigs	U.S. Transport Quality Assurance
1.	Those that are sick, injured, weak or disabled	Non-ambulatory severe injury	Non-ambulatory deep cuts	Not included in the text	Non-ambulatory severe injury	Unable to stand up and remain up	Unable to walk, significant injury, OIE guideline in document
2.	Those that are unable to stand unaided and bear weight on each leg	Fractures	Broken leg or unable to bear weight on two legs	Not included in the text	Difficulty walking	Not included in the text	Unable to walk Significant injury OIE guideline in document
3.	Those that are blind in both eyes	No Guidelines Published for Pigs					OIE Guideline in document
4.	Those that cannot be moved without causing additional suffering	Uterine prolapse Multiple joint arthritis	Not included in the text	Same as OIE unless instructed by a Vet	untreated necrotic prolapse	Uterine prolapse severe injury	Significant injury OIE guideline in document
5.	Newborn unhealed navel	Not Applicable for Sows and Boars					
6.	Pregnant animals which would be in the final 10% of their gestation period at planned time unloading	No guideline	Not included in the text	Sows last 10% of pregnancy (12 days)	No Guideline	Sows last 10% of pregnancy	OIE guideline in document
7.	Female traveling without young that had given birth within previous 48 h	No guideline	No guideline	Have given birth in the last week		No guideline	OIE guideline in document
8.	Those whose body condition would result in poor welfare because of expected climatic condition. (No explanation is included)	BCS 1 emaciated	BCS 1 emaciated	No guideline	BCS 1 emaciated	No guideline on BCS	OIE guideline in document No BCS score given

^1^ References for the different guidelines are [9,18,22,24,25,26]; ^2^ North American Meat Institute (Former name American Meat Institute).
